# Supplementary material for: Dysfunction of EAAT3 Aggravates LPS-Induced Post-Operative Cognitive Dysfunction
Source: Membranes (Basel). 2022 Mar 11;12(3):317. doi: 10.3390/membranes12030317 (PMC8951453; doi:10.3390/membranes12030317)
Supplement: Supplementary file 1 [file membranes-12-00317-s001.zip › Supplementary materials.pdf]

Supplementary material 1

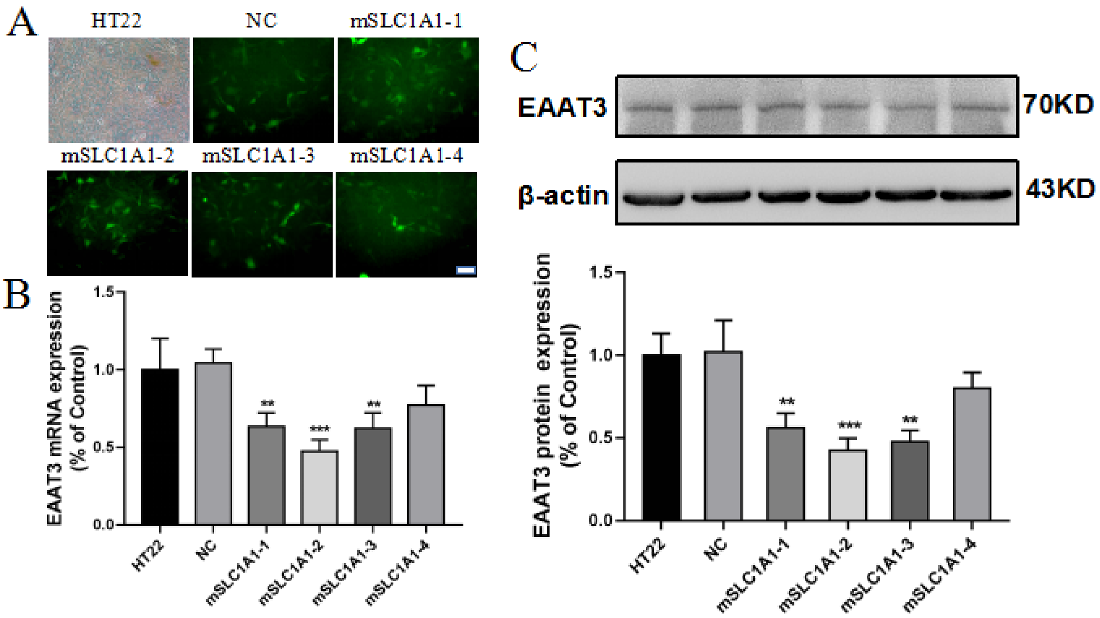

Suppl. Figure S1. Screening results of rAAV infecting H22 cell line. (A–C) Representative images were taken with ×200 fluorescent inverted microscopy 72 hours after transfection (**A**), and EAAT3 relative expression in cells transfected with different rAAV by mRNA (**B**) and western blot (**C**) assays (n = 3). $^{**}p < 0.01$, $^{***}p < 0.001$, vs. HT22. rAAV (recombinant adeno-associated viral); NC(rAAV-shRNA-NC); mSLC1A1-1～4(rAAV-shRNA-SLC1A1-1～4). Bar = 50 μm.

Supplementary material 2

Materials and Methods

Adult (3 months, weighing 22–25g) and old (21 months, weighing 28–36g) male C57BL/6 mice were obtained from Beijing SPF Animal Technology Company (Beijing, China. Permit Number: SCXK1026-0002).

Six adult and six old mice were randomly selected as Adult and Old groups. Animals were decapitated, and their hippocampi were removed for western blot analysis. western blot was carried out as previously described in the text.

Result

Hippocampal EAAT3 protein expression was significantly decreased in old mice

To determine whether there was any difference in the expression of EAAT3 in the hippocampus of adult and old mice, we measured the expression of total protein and membrane protein in the hippocampus of mice in the Adult and Old group by western blot. The results showed that EAAT3 expression of total protein ($^{**}p < 0.01$) and membrane protein ($^{**}p < 0.01$) in the hippocampus of the Old group was significantly lower than that in the Adult group (**Suppl. Figure S2**).

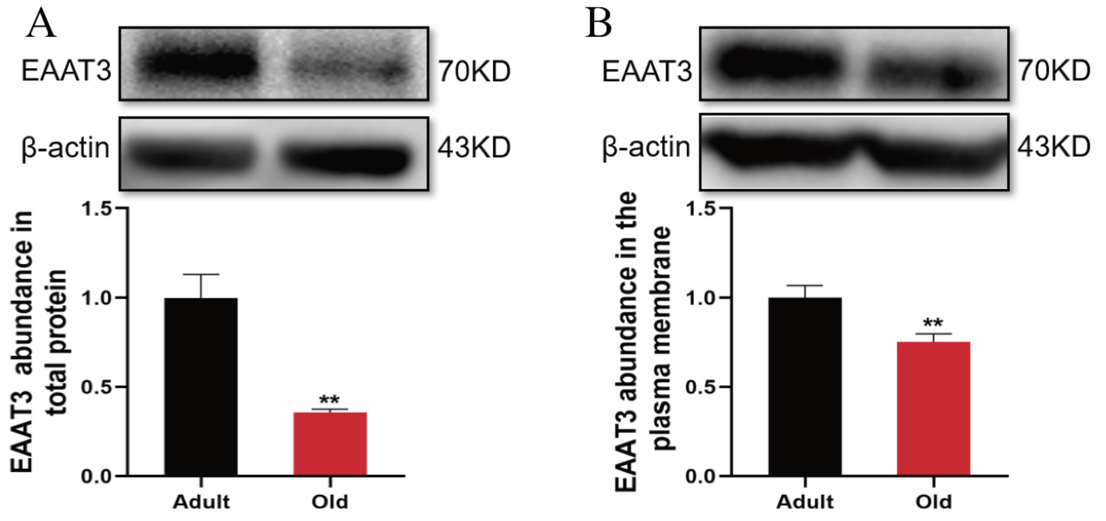

Suppl. Figure S2. Expression of EAAT3 in old mice was significantly lower than in adult mice. (A,B) Expressions of EAAT3 total protein (**A**) and membrane protein (**B**) in the hippocampus of old mice significantly decreased compared to those of adult

mice. Data are expressed as mean ± SEM (n = 6). ** $p < 0.01$. Excitatory amino acid transporter 3 (EAAT3); SEM, standard error of mean.

Supplementary material 3

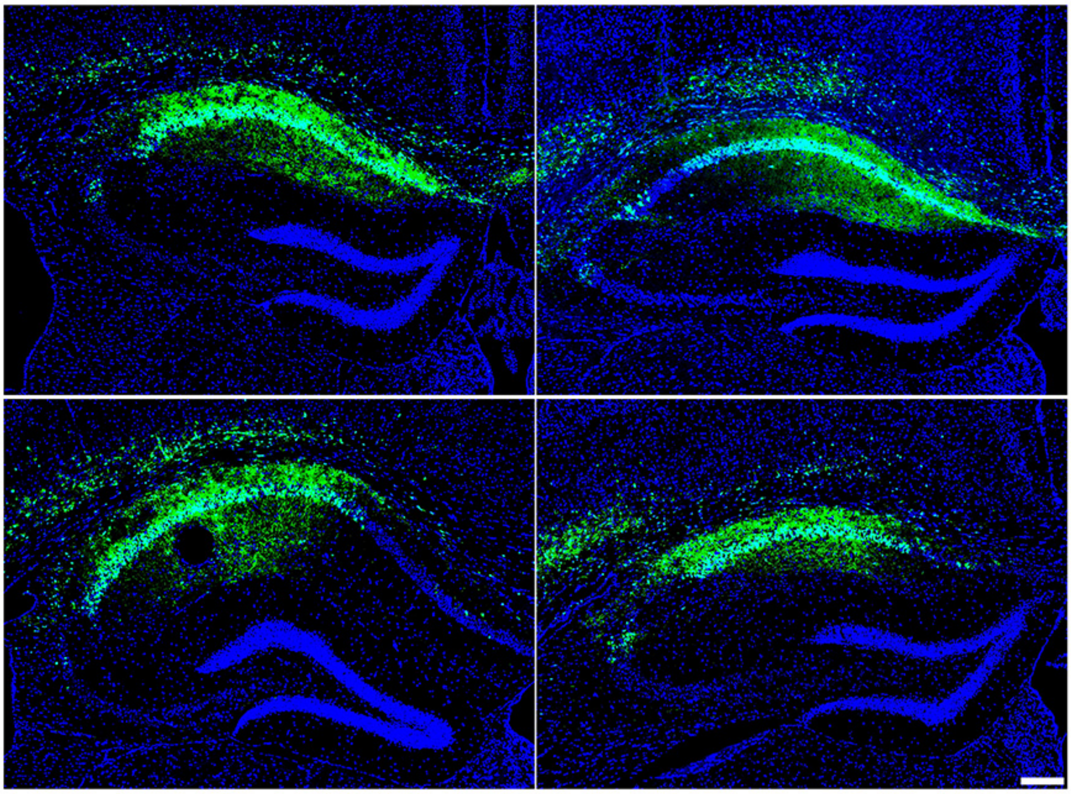

Suppl. Figure S3. The rAAV spreads to the entire CA region of the hippocampus 21 days after adult mice receiving the hippocampal injection. GFP indicates cells in the hippocampus transduced with RNAi. rAAV (recombinant adeno-associated viral); GFP (green fluorescent protein); RNAi (RNA interference vector). Bar = 100 µm.
